# Reply: Extracellular vesicle-mediated signalling in human rescue *in vitro* maturation: methodological and mechanistic considerations

**DOI:** 10.1093/hropen/hoag050

**Published:** 2026-05-28

**Authors:** Sofia Makieva, Mara D Saenz-de-Juano, Carmen Almiñana, Stefan Bauersachs, Sandra Bernal-Ulloa, Min Xie, Ana G Velasco, Natalia Cervantes, Maike Sachs, Susanne E Ulbrich, Brigitte Leeners

**Affiliations:** Department of Reproductive Endocrinology, University Hospital Zurich, Zurich, Switzerland; Animal Physiology, Institute of Agricultural Sciences, ETH Zurich, Zurich, Switzerland; Department of Reproductive Endocrinology, University Hospital Zurich, Zurich, Switzerland; Functional Genomics Group, Institute of Veterinary Anatomy, University of Zurich, Zurich, Switzerland; Animal Physiology, Institute of Agricultural Sciences, ETH Zurich, Zurich, Switzerland; Department of Reproductive Endocrinology, University Hospital Zurich, Zurich, Switzerland; Department of Reproductive Endocrinology, University Hospital Zurich, Zurich, Switzerland; Department of Reproductive Endocrinology, University Hospital Zurich, Zurich, Switzerland; Department of Reproductive Endocrinology, University Hospital Zurich, Zurich, Switzerland; Animal Physiology, Institute of Agricultural Sciences, ETH Zurich, Zurich, Switzerland; Department of Reproductive Endocrinology, University Hospital Zurich, Zurich, Switzerland

Dear Editor,

We thank Dr Sun (Sun, 2026) for the interest in our work (Makieva *et al.*, 2026) and for the opportunity to further discuss follicular fluid extracellular vesicles (ffEVs)-mediated regulation of human oocyte maturation. We view this correspondence positively as increased discussion around extracellular vesicles (EVs) in human *in vitro* maturation (IVM) is valuable for the field, particularly given the limited availability of human data. The points raised are relevant and allow clarification of methodological choices and interpretation.

With respect to ffEVs isolation, we agree that differential ultracentrifugation enriches rather than fully purifies ffEVs populations. This limitation is widely recognised and consistent with commonly used approaches in EVs research. The MISEV2023 guidelines state that no single separation method is required or prohibited and that commonly used techniques, including differential ultracentrifugation, yield heterogeneous EVs-containing preparations (Welsh *et al*., 2024). The guidelines recommend interpreting such fractions as EVs-enriched rather than purified vesicles and emphasize transparent reporting.

In our study, ffEV preparations were characterised by particle sizing, transmission electron microscopy, and detection of canonical EVs markers, and functional effects were observed following supplementation (Makieva *et al.*, 2026). Similar isolation and characterisation strategies have been used in our previous work involving EVs derived from other biological sources (Giacomini *et al.*, 2021; Makieva *et al.*, 2024; Saenz-de-Juano *et al.*, 2025). Several studies isolating EVs from follicular fluid have also employed differential ultracentrifugation-based workflows (Asaadi *et al.*, 2021; Neyroud *et al.*, 2022; Bortot *et al.*, 2025; Chang *et al.*, 2025). Our findings should therefore be interpreted as reflecting the biological activity of an EVs-enriched follicular fluid fraction.

Regarding pooling of follicular fluid samples, this strategy was intentionally used to reduce donor-specific variability and obtain sufficient material for functional assays and single-oocyte proteomics. Human rescue *in vitro* maturation (rIVM) experiments are inherently limited by sample availability, and pooling enables detection of reproducible biological effects. Importantly, all ffEVs used for supplementation originated from follicles containing mature oocytes, preserving biological relevance. Future studies addressing donor-specific ffEVs populations will be important to explore heterogeneity; however, pooling does not affect the interpretation of the observed maturation-associated effects. Pooling strategies are commonly applied in EVs research when material is limited. For example, we previously analysed pooled EVs derived from embryonic secretomes with the same genetic diagnosis in the context of preimplantation genetic testing. Despite pooling, this approach enabled identification of informative diagnostic biomarkers (Makieva *et al.*, 2024). These considerations support the use of pooling as a pragmatic strategy for exploratory human EVs studies.

We agree that polar body extrusion reflects nuclear maturation and does not fully represent developmental competence. Our study was not designed to assess fertilisation or embryo development, and we did not interpret our findings as evidence of improved competence. Instead, we evaluated multiple maturation-associated features, including nuclear maturation, single-oocyte proteomic changes, and ultrastructural organisation. ffEVs supplementation was associated with altered abundance of proteins linked to oocyte maturation and redistribution of endoplasmic reticulum–mitochondria complexes, recognised indicators of cytoplasmic maturation. These complementary observations support the interpretation that ffEVs influence maturation-related processes beyond nuclear progression. This distinction is particularly relevant in rIVM, where denuded oocytes lack somatic support and frequently exhibit compromised cytoplasmic maturation and developmental competence (Coticchio *et al.*, 2025). Accordingly, studies in rIVM commonly rely on nuclear maturation or intermediate cellular endpoints rather than embryo development. For example, Esbert *et al.* (2024) assessed chromosome segregation and euploidy in rIVM oocytes reaching metaphase II, reflecting the practical limitations of assessing developmental competence in human oocytes.

Assessment of developmental competence in human oocytes is also constrained by ethical and regulatory considerations. Fertilisation of donated human oocytes exclusively for research purposes is subject to strict approval processes and is not routinely feasible, particularly for rIVM material derived from clinical cycles. Parthenogenetic activation may provide an alternative functional model; however, parthenotes do not recapitulate normal fertilisation or biparental embryo development and therefore have inherent limitations. For these reasons, our study focused on maturation-associated phenotypes that can be assessed within ethical and practical constraints of human research.

The observation that HAS1 increased in supplemented oocytes despite not being detected in the ffEVs proteome supports an indirect mechanism of EVs-mediated signalling rather than simple transfer of vesicular proteins. EVs may influence recipient cells through downstream signalling triggered by internal cargo or surface interactions. Recent conceptual work has emphasised that EVs signalling may occur through multiple modes, including even surface binding without internalisation, challenging a simple cargo-transfer model (Chrzanowski and Wolfram, 2026). In this context, the increase in HAS1 may reflect downstream cellular reprogramming triggered by ffEVs exposure.

Our study was designed as a first human proof-of-concept to test whether ffEVs can provide biologically relevant support to rIVM. Immature oocytes are routinely obtained during stimulated IVF cycles but frequently remain in suboptimal culture conditions with limited developmental potential. rIVM therefore represents an attempt to improve material that is already available but currently underutilised. However, rIVM is particularly challenging because immature denuded oocytes are removed from the follicular microenvironment and lack the somatic support required for coordinated nuclear and cytoplasmic maturation. Identifying approaches that partially restore this signalling environment is therefore of interest. Supplementation with ffEVs is biologically plausible, as these vesicles are native components of follicular communication. We do not suggest that such an approach can reverse intrinsic oocyte limitations. Rather, the aim is to support maturation-associated processes in a population of oocytes that is often considered compromised but is nevertheless routinely retrieved, cultured, inseminated, or cryopreserved. Our approach therefore seeks modest improvement of maturation biology rather than transformation of inherently poor-quality oocytes. These findings may also inform future work aimed at improving IVM strategies more broadly, although this was not the primary scope of this study.

In summary, we appreciate the constructive discussion. We believe our findings provide initial human evidence that follicular fluid EVs modulate maturation-associated processes and establish a foundation for further mechanistic and translational studies.
